# Digital media engagement and its relationship with depression severity

**DOI:** 10.3389/fpsyg.2026.1926446

**Published:** 2026-10-20

**Authors:** Areeba Syed, Marek Preiss, Dominika Grygarová, Zuzana Podaná, Jiří Horáček

**Affiliations:** 1 National Institute of Mental Health, Klecany, Czechia; 2 First Faculty of Medicine, Department of Psychiatry, Charles University, Prague, Czechia; 3 Department of Psychology, University of New York in Prague, Prague, Czechia; 4 Third Faculty of Medicine, Charles University, Prague, Czechia; 5 Faculty of Arts, Charles University, Prague, Czechia

**Keywords:** depression, emotional impact, interactive content, media exposure, media stress, psychological distress, readers' comments

## Abstract

**Introduction:**

Throughout the pandemic, social media became the new newspaper, and the widespread information created a vulnerable state for the user. The study's goal is to determine the presence of depression in the Czech Republic's local population as well as how the use of media correlates with depression in the Czech population.

**Methodology:**

A web-based interviewing technique was employed. A longitudinal study method was employed, and the data were collected in 5 waves. In each wave, the population varied in number: 2,192 (2020), 2,164 (2021), 1,717 (2022), 1,707 (2023), and 2,072 (2025), and listwise 1,120 participants participated in all 5 waves. The trend analysis of depression using PHQ-5 and MUQ was calculated for the years (2020–2023 & 2025).

**Results:**

Total PHQ mean scores rose progressively from 7.41 (*SD* = 3.02) in 2020 to 8.60 (*SD* = 3.58) in 2025. Correlation analyzes revealed distinct, wave-dependent patterns across media engagement dimensions. Component 2 (Interactive Content and Reader Comments) demonstrated the most consistent positive correlation with depressive symptoms across all waves (*r* = 0.233 to 0.305, *p* < 0.01, moderating it *r* = 0.147, *p* < 0.01 in 2025). Component 3 (Emotional Impact and Media Stress) was positively correlated with depression during active crisis years (*r* = 0.069 to 0.125, *p* < 0.01 from 2020 to 2023) but exhibited a negative correlation in 2025 (*r* = −0.268, *p* < 0.01). Component 1 (Economic Reporting and Serious News) maintained weak or non-significant associations throughout the study period (*r* = 0.011 to 0.055).

**Conclusion:**

The results indicate that Emotional reactivity is significantly correlated with media-related depression, while interactive content has a variable multiplier effect, and analytical news has a low direct effect. Public health interventions should prioritize emotional regulation, media literacy, and affective platform design to curb population-level mental health declines.

## Introduction

### Mental illness and media

The media has become an integral part of everyday life in this digital age, fundamentally affecting the way people see themselves and the world around them. Media is a powerful tool for raising awareness and building connections, but it also has a complex and often negative effect on mental health, especially concerning depression (Rozanov and Rutz, 2021; Chao et al., 2020; Propper et al., 2007). An estimated 3.8 billion people were users of social media by the year 2020 (Kemp, 2020). The rapid adoption of the technology presented a new opportunity for addressing the gaps in mental healthcare and helped it become more accessible (Feijt et al., 2020). Numerous people utilize social media to share their own psychological issues and listen to other people's experiences, forming an empathetic community that acts as a safe space for seeking help, challenging stigma, and developing healthy coping mechanisms (Berry et al., 2017; Hua et al., 2025; Skaik and Inkpen, 2020). And recent research advocates for leveraging social media's popularity to enhance therapeutic delivery (Ahmed et al., 2022).

Social media, however, plays a dual role. On the one hand, it connects individuals who require support to practitioners, thereby making grounds for awareness and aiding in stigma reduction (Grygarová et al., 2022). But on the other hand, there is no certainty regarding the audience consuming the content, and the inability to create a targeted response for specific groups makes the attainment of positive results uncertain. Overwhelming reach with no targeted consumers, and a lack of accountability, where sometimes the information providers are not even professionals, can cause significant problems. Not every piece of content on social media is factual or created by experts in the field. A WHO-commissioned review of reviews estimated that up to 28.8% of health content on social media is misleading, with platforms like Twitter, Facebook, and YouTube acting as primary vectors of health-related misinformation. As per a systematic review of 69 studies, up to 87% of posts on certain health topics constituted misinformation. This is made possible structurally by the lack of editorial gatekeeping; any user, regardless of experience, may publish and spread claims, and the system lends priority to engagement rather than authenticity (Nascimento et al., 2022; Pennycook and Rand, 2022; Rocha et al., 2021).

Additionally, the cogency of the online support programs is often a catalyst for the users' internal psychological state. The emotional self-regulation is mostly central to the digital wellness theory, as the impact of it depends on the individual's psychological wellbeing. Chen et al. (2026) note that the suppressed emotions are at risk for internet addiction and depressive symptoms, whereas the cognitive reappraisal acts as a protector of mental health. This shows that mental health outcomes are determined by how individuals cognitively process digital stimuli. Additionally, the media has been found to promote the romanticization of pain, reinforce prejudices, and exacerbate feelings of loneliness and inadequacy (Shrestha, 2018; Rick, 2016). Thus, practitioners must navigate the persistent and debated risks these platforms pose to user wellbeing (Orben and Przybylski, 2019).

### Negative effects of media on mental health

Emotional arousal and a sense of uncertainty are frequently triggered by media coverage that highlights threat, ambiguity, or sensationalist narratives (Kesner et al., 2025). In various studies, emotional and negative portrayals of war and disease-related news have been found to induce negative psychological effects among the population. Excessive exposure to stressful information caused trauma in the Czech population after March 2020, as reported by Trnka and Lorencova (2020). Due to their isolation and limited opportunities to express their concerns and feelings to others, the demographic that was most impacted was seniors. The Czech mass media released hundreds of stories about COVID-19-infected people from the non-vulnerable portion of the global population who either died or experienced highly rare or exceptionally serious health effects, to reach a wider audience (Grygarová et al., 2022). During the initial weeks of the COVID-19 outbreak, many of these media messages were delivered in an extremely emotive way, which exacerbated traumatic emotions, worries, and discomfort (Dong and Zheng, 2020).

According to an increasing number of studies, the psychological effects of war are felt by people all around the world due to ongoing media coverage (Kurapov et al., 2023; Juríčková et al., 2025). People who live in war-torn countries like Ukraine may become overly obsessed with the future, either in the form of effects caused by the threat they are facing or imagining a fantastical reality separate from the disparity they are currently facing (Kesner et al., 2025).

According to Malecki et al. (2023) and Silver et al. (2013), this media-transmitted trauma can cause serious secondary trauma and stress reactions even in those who are not in immediate danger. This phenomenon, which is frequently referred to as “vicarious trauma,” presents a serious risk of indirect traumatization, a condition in which people are indirectly affected psychologically by being exposed to the pain of others (Figley, 1995). Li et al. (2020) stated that the long-term decline in mental health indicates that continuous media exposure serves as a conduit for vicarious traumatization. The proliferation of channels and 24-h coverage of news in the digital age guarantees constant exposure to violent images and stories of anguish caused by combat (Regnoli, 2025). Liu and Liu (2020) on the other hand, indicated that COVID-19-related exposure is directly linked to vicarious traumatization of the populace. Depressive symptoms, which may arise because of indirect exposure to humanitarian distress through media, are central to these disruptions (Gottschick et al., 2023; Mottola et al., 2023).

Kesner et al. (2025) point out that traumatic news does not reflect most of the news stories shared on media networks and hypothesize that the current focus on vicarious psychological trauma has concealed the fact that ambiguity seems to have a significant role in mediating the complex interplay between media news consumption and its harmful effects on mental health. Boukes and Vliegenthart (2017) and De Hoog and Verboon (2020) concluded that even the exposure to normal, non-traumatic news has been associated with a negative emotional state and a negative impact on mental health. One of the processes that could cause these effects is rumination, whereby repeated exposure to distressing or ambiguous news can result in doubt and cognitive fixation. As a result, ambiguity becomes a significant psychological consequence of media-induced adversity rather than only a contextual aspect (Kesner et al., 2025).

The negative news coverage highlighting threat and ambiguity or sensationalist stories can create emotional arousal and uncertainty. This in turn creates a domino effect of negative thoughts. Ma and Liu (2026) determined rumination is moderately related to anxiety (*r* = 0.47) and depression (*r* = 0.46) and explains the most significant variance in depressed results among various social media variables.

Grupe and Nitschke (2013) further note that exposure to crisis-related material promotes negative self-referential thinking, which increases cortisol and fosters hypervigilance. Furthermore, chronically consuming ambiguous news from social media may exacerbate existing strains a person might be under (Cange et al., 2019; Nickerson et al., 2023). Contemporary digital media creates an environment of increased uncertainty, where habitual involvement and the fear of missing out (FOMO) contribute to problematic use and cognitive fatigue. Lee-Won et al. (2025) note that the “always-on” nature of these platforms ensures that the psychological system never fully “turns off,” causing a stress response designed for acute, temporary dangers to become chronically activated. According to Ottaviani (2018) and Kesner et al. (2025), this environment locks users in a cycle of obsessive monitoring and perseverative cognition, where concern and rumination serve as extended stressors that prolong physiological arousal long after the initial digital trigger has passed.

A framework for perseverative cognition is ideally suited to the effect of rumination and wear and tear on the trajectory of informational vigilance. Even when quick action is not feasible, repeated crisis monitoring can serve as a prolonged cognitive stressor that maintains physiological and emotional alertness (Ottaviani, 2018; Epel et al., 2018). Through rumination and emotion dysregulation, hopelessness is indirectly linked to intolerance of uncertainty, a fundamental characteristic of anxiety and depression (Grupe and Nitschke, 2013; Hernández-Posadas et al., 2023). Hoyt et al. (2022) found that the degree of media consumption is directly proportional to levels of anxiety; as inconsistent news reporting causes uncertainty and confusion, it is furthermore significantly associated with a rise in reported cases of depression. Even so, the relationship specifically between economic and serious news and depression is under-investigated, with no prior study investigating the relationship between these variables amongst the Czech population. Aiming to address this gap, we hypothesized that media component 1 (economic and serious news) is positively correlated with depression.

### Addiction to social media

Research suggests that addiction to various social media platforms can lead to depression among younger populations. This is illustrated by de Wit et al. (2011), who found that adults with major depressive disorder spent excessive amounts of free time on computers when compared to a healthy control group. However, in addition to the clinical definition of addiction examined by Chen and Shi (2026), the subjective perception of digital media stress may serve as a precursor to the development of depressive symptoms.

Prior research has found positive associations between Facebook use and depression in high school students (Pantic, 2014). Analysis of the data from a large database of over 19,000 subjects revealed a consistent pattern of findings that connects self-reported depression with higher media consumption (Block et al., 2014). For those who have positively reported depression, this media use was mostly concentrated on TV exposure and internet use. Twenge (2020) suggested that the increasing occurrence of disorders such as depression, especially among adolescents, is linked to characteristics of the technological environment. There is curiosity about how media consumption patterns might be connected to it from a neuromarketing approach. According to Vences et al. (2020) theory, neuromarketing plays a crucial role in creating emotional bonds with viewers on digital platforms. The literature serves as the baseline to associate that the media component 3 (Emotional Impact and Media Stress) will show a positive relationship with depression among the Czech population.

### Impact of social media interactivity

Another aspect is that anonymous comments play a vital role in the interaction. Through several interrelated mechanisms, negative comments can worsen depressive symptoms and threaten psychological wellbeing. According to Ibarra-Rovillard and Kuiper (2011), when someone is classified as depressed, listeners tend to respond negatively toward the comments, especially self-defeating ones, which make the recipients feel worse about themselves and the speaker. Low self-esteem, a fundamental characteristic of depression, is reinforced by this dual effect on social perception and self-evaluation (Zhou et al., 2020). Additionally, by encouraging people to take even neutral remarks negatively, such labeling exacerbates stigma and increases feelings of social isolation and rejection. Maladaptive cognitions, such as feelings of unworthiness or unlikability, are fundamental to cognitive-behavioral models of depression and help sustain depressive affect. They are further reinforced by hostile feedback.

Frequent exposure to critical or stigmatizing reactions can result in social disengagement, which diminishes chances for constructive interpersonal reinforcement and exacerbates depressive symptoms. People with high levels of cognitive impairment or trouble recognizing emotions seem to be especially susceptible to the depressing effects of online criticism; these effects may be amplified in digital situations where anonymity and quick broadcast magnify negative feedback. All things considered, stigmatizing social encounters serve as powerful stresses that can cause, prolong, or exacerbate depressive episodes by harming one's self-concept, fostering social disengagement, and reinforcing negative ideas (Marano et al., 2025). Considering this, we aimed to identify the relationship between media component 2 (Interactive content and reader comments) and depression, hypothesizing that people who have greater engagement with digital platforms tend to project higher depressive symptoms.

This hypothesis is aligned with the Response Styles Theory, according to which people who repeatedly focus on unpleasant feelings or news stories maintain their psychological arousal long after the original exposure (Nolen-Hoeksema, 2000). In a digital setting, this takes the form of “doomscrolling,” in which the user internalizes negative content through mental rehearsal in addition to initial consumption. As well as being a strong long-term predictor of internet addiction and depressed symptoms, this process hinders emotional recovery (Chen et al., 2026). The temporary stress of a news cycle starts to solidify into long-lasting depressive pathology when participants are unable to escape from distressing digital narratives cognitively.

Researchers are increasingly using the Allostatic Load Model to comprehend the long-term effects of this cycle (Lee-Won et al., 2025). The bodily and psychological “wear-and-tear” that develops when a person is subjected to frequent or ongoing stress is known as allostatic load. Although the human stress response is adaptive for short-term survival, years of digital stressors, from the COVID-19 pandemic (2020) to more recent geopolitical conflicts (2025), cause a persistent “over-activation” that drains a person's hope and psychological resilience (Chen and Shi, 2026).

This cumulative load implies that the effects of digital media are a developmental trajectory rather than just a “snapshot” response. According to recent longitudinal research, this wear-and-tear is accelerated by the loss of emotional control and the dependence on emotional suppression rather than cognitive reappraisal, which eventually manifests itself as self-reported depression (Chen et al., 2026).

Extensive literature focuses on digital media exposure as a singular behavioral process; however, there is a gap in critical research in relation to the tracking of different, multidimensional modalities of engagement with digital media over long periods of time relative to mental health outcomes. To overcome this constraint, the current research employs a unique five-wave longitudinal study design (2020–2023, 2025) to explore the synchronous tracking patterns of independent media components and depressive symptoms. Conceptually, this study is not just a screen-time study but provides a cognitive-behavioral-physiological approach to the overall issue. This distinction allows for a more complex understanding of the relationship between certain digital behaviors and psychological distress, as interactive engagement is separated from passive consumption. The main aim of this research is to explore the longitudinal patterns of depressive symptoms within the Czech population and to investigate the relationship between three different dimensions of media (Component 1 - Economic Reporting and Serious News, Component 2 - Interactive Content and Reader Comments, Component 3 - Emotional Impact and Media Stress) and self-reported depression over time.

#### Conceptualizing digital media engagement

In this study, Digital media engagement is defined in this study as a complex, multi-dimensional construct, rather than a simple screen time measure, and includes behaviors. It involves three different but interrelated components: exposure (how long and how often one is exposed to media), interaction (how actively they engage with media, e.g., create content, post or message), and emotional response (innermost psychological and affective experiences that result from their use of media, e.g., positive connection or negative comparisons). This multi-dimensional approach acknowledges both the observable, behavioral traces and the internal, psychological aspects of engagement with digital media, and offers a complete picture of users' observable and felt dimensions of engagement with the digital medium in their everyday lives.

### Framework

This study is multidisciplinary, combining cognitive, behavioral, and physiological approaches to create a coherent framework that connects digital media engagement with mental health. Actions of “doomscrolling” reinforce negative thinking patterns such as rumination, which continually interfere with functioning emotional regulation. This digital distress, if not managed to de-escalate, becomes a chronic psychological burden and then a chronic physiological burden. This cumulative wear and tear lead to higher allostatic load over time, causing structural changes to the body's stress response systems, and offers a clear pathway between compulsive use of digital devices and clinical symptoms of depression.

To investigate longitudinal trends in self-reported depression among the Czech population from 2020 to 2025, this study combines the psychological wear-and-tear model of consistent digital exposure with three distinct media components: economic/serious news (Component 1), interactive content and reader comments (Component 2), and emotional impact/media stress (Component 3).

Chronic rumination and negativity bias are fuelled by Component 1, which supplies the cognitive raw material these processes require, while Component 2 acts as an emotional catalyst that converts those stressors into existential threats. Altogether, these factors are significant with emotional dysregulation and erode psychological resilience. Consequently, a person becomes more likely to exhibit depressive symptomatology (Ibarra-Rovillard and Kuiper, 2011; Marano et al., 2025).

According to the concept, doomscrolling, heightened threat perception, ambiguity associated with media, emotional overload, and rumination are all fuelled by consumption of media, which eventually lead to depressive symptoms. Formally referred to as allostatic overload, these reactions result in psychological wear-and-tear when they are maintained by the “always-on” character of contemporary media (Lee-Won et al., 2025).

This framework suggests that media use has a cascading longitudinal impact on depression (See Figure 1). Whereas Component 2 (Interactive Content and Reader Comments) is an affective amplifier, enhancing the perceived personal danger by employing emotionalized social cues, Component 1 (Economic Reporting and Serious News) provides the initial cognitive content which activates systematic thinking. Component 3 (Emotional Impact and Media Stress), which serves as the psychological “bridge” to clinical outcomes, is the product of these inputs taken together.

**Figure 1:**
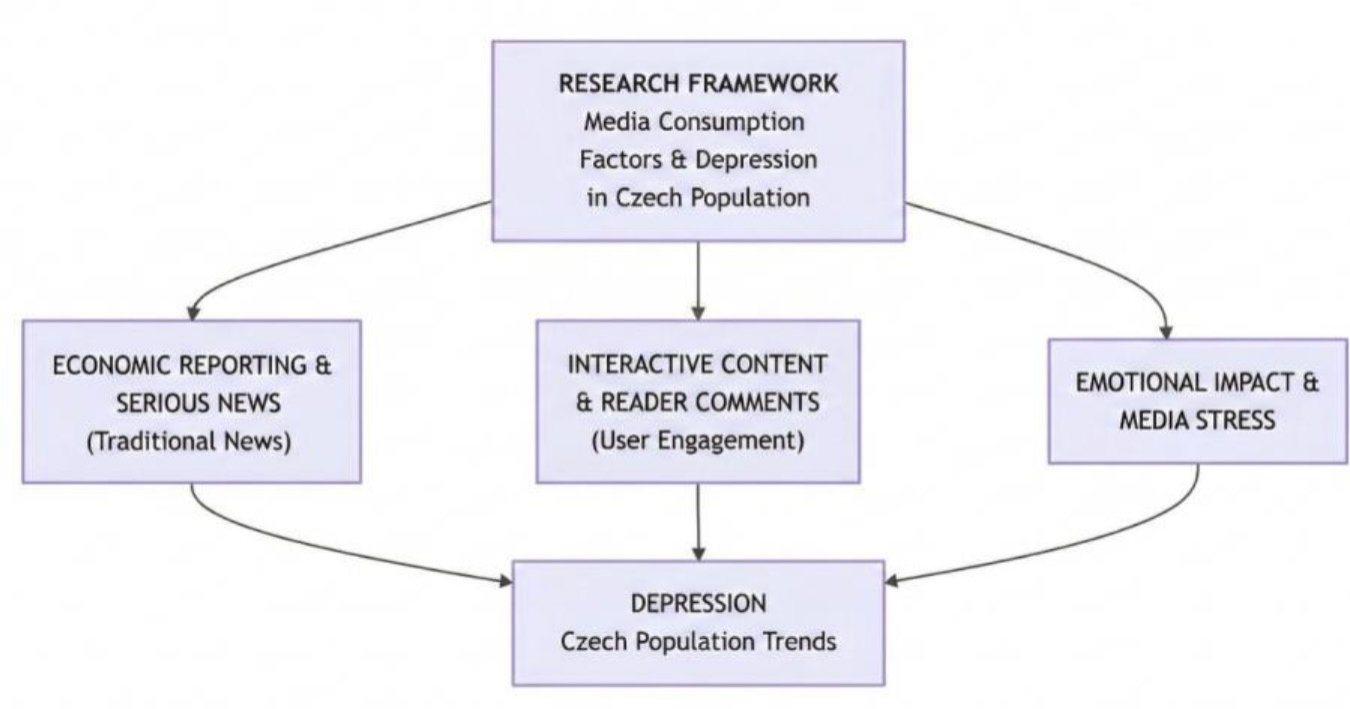
Framework of media and depression.

## Methodology

This study is a longitudinal, five-wave panel study design; the representative cohort was attained from the Czech National Panel, and a standardized computer-assisted web interviewing technique was conducted (Grygarová et al., 2022). Participants were drawn from all over the Czech Republic. Still, the majority lived in the capital city of Prague (Hlavní město Praha; 27.9%), followed by the Moravian-Silesian Region (Moravskoslezský kraj; 10.7%) and the South Moravian Region (Jihomoravský kraj; 9.7%). This ensured that the participants were from a variety of geographical areas. Data was gathered over 5 years, from 2020 to 2023 & 2025, in five phases. The population varied in number (Table 1): 2,192 (2020), 2,164 (2021), 1,717 (2022), 1,707 (2023), and 2,072 (2025) from which 1109 participants were consistent throughout 5 waves. The observed changes in the number of participants with valid data over the five time points are typical of the natural fluctuation that occurs in longitudinal survey designs, such as item non-response, waves of participant attrition, and re-engagement in later waves. Due to the listwise deletion used in the reliability and factor procedures, which provided data integrity per wave, the number of participants within each wave varied, as participants with missing responses on any one of the indicators were not included for that respective wave. The trend analysis of depression and media impact was assessed using PHQ-5 and MUQ for the years (2020–2023 & 2025) (See Table 1). The statistical analysis was done using IBM SPSS Statistics (Version 29.0). Pearson correlation models were used to examine the relationship between the three standardized media separately and the depressive symptoms at each discrete time point (2020-2023, 2025). Instead of assuming a static trajectory (longitudinal approach), this wave-by-wave associative approach was chosen to reflect possible changes in the strength and direction of these connections across different time perspectives.

**Table 1:** Longitudinal sample distribution, valid responses, and retention rates across waves (2020–2025).

Wave	Data collection year	Valid responses (*N*)	Missing responses (*n*)	Proportion of baseline sample (%)	Retention of total pool (%)
Wave 1	2020	2,192	905	100.0%	70.8%
Wave 2	2021	2,164	933	98.7%	69.9%
Wave 3	2022	1,717	1,380	78.3%	55.4%
Wave 4	2023	1,707	1,390	77.9%	55.1%
Wave 5	2025	2,072	1,025	94.5%	66.9%

Listwise *N* = 1,109.

### Depression

The severity of depression during the previous 2 weeks was assessed using the 8-item Patient Health Questionnaire Depression Scale (PHQ-8; Kroenke et al., 2009). A shortened version of the 5-point PHQ was utilized to reduce the number of items for this study (See Table 2). The shortened version was utilized because this study was a longitudinal study, and part of a large epidemiological study, and the PHQ-5 was brief enough to effectively screen thousands of individuals for depression symptoms without creating survey fatigue. The Cronbach's alpha coefficient was excellent across waves (α = 0.865 in 2020, α= 0.869 in 2021, α = 0.879 in 2022, α = 0.891 in 2023, α = 0.891 in 2025), and the internal consistency of the scale across the two waves was strong. The concurrent correlations with external measures were quite high, and the factor structure was confirmed across time intervals, thus supporting construct validity.

**Table 2:** 5 Point patient health questionnaire.

Czech		English
PHQ1	Měl/a jsem malý zájem nebo potěšení z věcí, které dělám.	I had little interest or pleasure in the things I do.
PHQ2	Měl/a jsem pocit, že jsem na dně, pocit deprese nebo beznaděje.	I felt like I was at the bottom, feeling depressed or hopeless.
PHQ3	Měl/a jsem potíže s usínáním, prerušovaným spánkem nebo s prílišným spaním.	I had trouble falling asleep, staying asleep, or sleeping too much.
PHQ4	Měl/a jsem pocit únavy nebo málo energie.	I felt tired or had low energy.
PHQ5	Neměl/a jsem chut k jídlu nebo se naopak prejídal/a.	I had no appetite or, conversely, I overate.

### Media

The Media Use Questionnaire (MUQ) was developed to track 5 key indicators of media (See Table 3) consumption over the previous 2 weeks (Grygarová et al., 2022). The self-reported frequency of following different news platforms and categories (e.g., print newspapers, public service television and radio, mainstream news websites, alternative news websites, YouTube, and mobile applications) was used to measure media use. To find underlying trends in media consumption and media effect, Principal Component Analysis (PCA) extracted 3 components. The largest factor loadings (absolute value ≥0.40) of the items were used to allocate them to components.

**Table 3:** Media components derived from the media use questionnaire.

Item code	Czech original	English translation
koment_n3	Čtete si komentáře čtenářů pod články na zpravodajských portálech?	Do you read readers' comments under articles on news websites?
medi2	Sledování zpráv v médiích | Veřejnoprávní média – Česká televize a Český rozhlas	Following news in the media | Public service media – Czech Television and Czech Radio
rub13_n6	Politické a ekonomické zpravodajství	Political and economic news coverage
medi16_n6	Sledování zpráv v médiích | Zpravodajské weby jako iDNES, Novinky, Aktuálně, iHNED nebo tištěné deníky jako Hospodářské noviny, Právo, Deník, MF DNES	Following news in the media | News websites such as iDNES, Novinky, Aktuálně, iHNED, or printed newspapers such as Hospodářské noviny, Právo, Deník, MF DNES
medi15_n6	Sledování zpráv v médiích | Sociální sítě jako Facebook, X (dríve Twitter), Instagram a příspěvky na nich	Following news in the media | Social media such as Facebook, X (formerly Twitter), Instagram, and posts on these platforms
dome1	Dopad médií | Informace, které se dozvím ve zpravodajství v médiích, jsou častým tématem mých úvah a přemýšlení v každodenním životě.	Media impact | Information I learn from news media is a frequent topic of my thoughts and reflections in everyday life.
dome7	Dopad médií | Cítím se ve stresu, když se ze zpráv v médiích dozvídám o nejrůznějších příbězích a situacích.	Media impact | I feel stressed when I learn from news media about various stories and situations.
podro_n6	Pokud čtete zprávy v tištěných médiích nebo na zpravodajských portálech, jak podrobně je čtete?	If you read news in printed media or on news websites, how thoroughly do you read it?

Principal Component Analysis (PCA) with varimax rotation was applied across waves to extract three underlying media components: *Component 1 (Economic Reporting and Serious News):* Following political/economic news, mainstream news websites, print papers, and thorough news reading. *Component 2 (Interactive Content and Reader Comments):* Reading user comments under online news articles and news engagement on social media platforms (Facebook, X/Twitter, Instagram). *Component 3 (Emotional Impact and Media Stress)*: Reporting that news media is a frequent topic of daily reflection and experiencing personal stress when learning about news stories. The suitability of the data for Principal Component Analysis (PCA) was confirmed across all time points, yielding Kaiser-Meyer-Olkin (KMO) measures of 0.703 (2020), 0.745 (2021), 0.756 (2022), 0.758 (2023), and 0.740 (2025), with consistently significant Bartlett's Tests of Sphericity (χ^2^(28) = 2, 057.60 in 2020; χ^2^(28) = 2, 256.86 in 2021; χ^2^(28) = 2, 450.56 in 2022; χ^2^(28) = 2, 498.06 in 2023; χ^2^(28) = 2, 639.25 in 2025; all *p* < 0.001).

The ordinal response scale recorded usage frequency in six categories: several times per day (more than twice), once or twice per day, Several times per week, about once per week, less often, and never. Standardized (z-score) values of the individual items weighted by their corresponding factor loadings were used to compute component scores. Stronger support for the corresponding media consumption pattern or media-related response was indicated by higher component scores.

Exposure to media channels was assessed using the question “How often have you watched/read/listened to news in the following media channels in the past 2 weeks?” and was measured using the 6-point Likert scale (Several times a day (more than twice), Once or twice a day, Several times a week, About once a week, Less often, Never). None of these clusters included several media outlets (e.g., “Nova TV/Prima TV/ Barrandov TV”). Participants indicated how often they engaged with 12 media clusters, which encompassed a total of 34 media outlets (e.g., “NOVA TV/Prima TV/ Barrandov TV” grouped under one cluster).

## Results

The results were calculated using the SPSS software. Descriptive statistics were employed to analyse the demographic details (Table 4) of the individuals.

**Table 4:** Demographic information.

Variable	Type	*N*	%
Sex	Male	1,272	50.3%
	Female	1,256	49.7%
Religion	Without religious faith	1,489	58.9%
	Christianity—Catholic	375	14.8%
	Christianity—Protestant, Hussite, or other church	82	3.2%
	Islam	3	0.1%
	Judaism	5	0.2%
	Believer, but without a specific denomination	270	10.7%
	Other	61	2.4%
	Missing	243	9.6%
Education	Basic education	146	5.8%
	High School without graduation	746	29.5%
	Full Secondary education	912	36.1%
	University	724	28.6%
Region	Capital City of Prague	706	27.9%
	Central Bohemian region	236	9.3%
	South Bohemian region	108	4.3%
	Pilsen region	111	4.4%
	Karlovy vary region	47	1.9%
	Ústí nad Labem region	172	6.8%
	Liberec region	99	3.9%
	Hradec Králové region	108	4.3%
	Pardubice region	98	3.9%
	Vysočina region	99	3.9%
	South Moravian region	246	9.7%
	Olomouc region	115	4.5%
	Zlín region	113	4.5%
	Moravian-Silesian region	270	10.7%

The mean PHQ total scores rose progressively from 7.41 to 8.60 between 2020 and 2025, indicating a slow increase in depression symptoms in the general population (See Table 5). Although the annual rise in depression was slight, the steady upward trend suggests a concerning trend in mental health that warrants further research.

**Table 5:** PHQ mean differences 2020–2023 & 2025.

Wave/Year	Mean	Std. deviation	*N*
PHQ2025	8.604	3.578	2,072
PHQ2023	7.891	3.391	1,707
PHQ2022	7.641	3.225	1,717
PHQ2021	7.593	3.238	2,164
PHQ2020	7.413	3.024	2,192

Within the Pearson correlation model, three standardized media components were added as correlates to investigate the relationship between the standardized media components and depressive symptoms in the waves 2020–2023 and 2025. The longitudinal tracking shows that the relations between the different media elements and the depression symptoms, as assessed by the Patient Health Questionnaire (PHQ), differ importantly over time and do not consistently converge to any linear or path direction. In particular, the intensity of these associations varies each calendar year, and some of the components move in tandem with elevated or depressed levels of symptoms in the corresponding year. These results suggest context-sensitive and synchronous shifts between different media engagement profiles and mental health states rather than a one-sided relationship.

### Component 1: economic reporting and serious news

Throughout the study period, there was little to no correlation between depressive symptoms (measured by PHQ scores) and the intake of serious news and economic reporting (Component 1). No statistically significant link was found for 2020 (*r* = 0.011, *p* > 0.05), 2021 (*r* = 0.020, *p* > 0.05), or 2023 (*r* = −0.021, *p* > 0.05). In 2022, there was a little but noteworthy positive connection (*r* = 0.055, *p* < 0.05). By 2025, the relationship shifted to a statistically significant negative association (*r* = −0.104, *p* < 0.001) (See Table 6), indicating that higher consumption of serious news was modestly associated with lower PHQ-5 scores at the final wave.

**Table 6:** Media consumption and depression (PHQ-5): correlation matrix.

Media component	2020	2020 Sig.	2021	2021 Sig.	2022	2022 Sig.	2023	2023 Sig.	2025	2025 Sig.
Serious news & economic reporting	0.011	ns	0.020	ns	0.055	^*^	−0.021	ns	−0.104	^**^
Reader comments & interactive content	0.233	^**^	0.275	^**^	0.256	^**^	0.305	^**^	−0.276	^**^
Emotional impact & media stress	0.069	^**^	0.125	^**^	0.094	^**^	0.110	^**^	0.150	^**^

*N* = 2,192 (2020), 2,164 (2021), 1,717 (2022), 1,707 (2023), and 2,072 (2025). ns, non-significant (*p* > 0.05). ^*^ *p* < 0.05, ^**^ *p* < 0.01.

### Component 2: interactive content and reader comments

Component 2 (Reader Comments and Interactive Content) had the strongest and most reliable correlation with measures of mental health. This component showed a positive correlation throughout all years recorded, increasing from *r* = 0.233 in 2020 to *r* = 0.305 (*p* < 0.05) in 2023. In 2025, however, this relationship inverted significantly, emerging as a negative correlation (*r* = −0.276, *p* < 0.001). These findings suggest that active engagement with interactive features and news comments transitioned over time from a correlate of higher depressive distress during acute crisis phases to a protective or buffering factor in the post-crisis period.

### Component 3: emotional impact and media stress

It stands for perceived media stress and the emotional impact of media, which showed a steady but erratic correlation with PHQ scores. Component 3 had a steady positive correlation from 2020 to 2023, reaching its highest point in 2021 (*r* = 0.125, *p* < 0.05). This suggests that there was a substantial correlation between greater depressed symptoms and higher levels of stress associated with the media during this time. This association remained positive and highest observed (*r* = 0.150, *p* < 0.001) in 2025 (See Table 6). This implies that by 2025, there will have been a substantial descriptive shift in the type of media stress or in the population's psychological resilience in relation to emotionally charged media material.

The results overall suggest that interactive engagement (Component 2) and emotional stress reactivity (Component 3) are better correlates of depressive symptoms than is routine content consumption (Component 1).

## Discussion

This study aimed to investigate the trends of depression among the Czech population, and the findings show that the population's trends of depressive symptoms have been steadily rising over the past 5 years, according to the PHQ-5 score analysis (2020–2023 & 2025). The mean scores shifted upwards from 7.41 in 2020 to 8.60 in 2025. The sample size guarantees the significance of the study, even with the variation of the sample size over the different waves. The standard deviation showed that the distribution of the severity among the Czech population remained unchanged.

Over five waves (2020–2025), this longitudinal study looked at the changing association between three different media-consumption components and depression symptoms (PHQ). Our main conclusion is that there was no media behavior that had a consistent psychological impact. Rather, throughout time, there was a major functional restructuring of each component: Component 1 (Economic Reporting and Serious News), Component 2 (Interactive Content and Reader Comments), Component 3 (Emotional Impact and Media Stress). These findings support a dynamic, context-sensitive model of digital media effects, in which identical behaviors may transition from adaptive to maladaptive based on the stage of an extended crisis, with rumination serving as the principal cognitive mechanism and wear-and-tear (allostatic load) as the fundamental biological and psychological process.

The relationship between media use and depression symptoms was found to have a dynamic nature over the 5-year duration of the study, as opposed to one that was fixed. Shannon et al. (2022) and Orben and Przybylski (2019) note a growing consensus that content, intensity, context, and personal vulnerability have a significant impact on the effects of digital media. Big-picture investigations of digital technology and mental health have found varied and often small relationships instead of a direct negative or positive impact (Coyne et al., 2019; Orben and Przybylski, 2019), although problematic social media use demonstrates moderate relationships with depression, anxiety, and stress (Ahmed et al., 2022; Shannon et al., 2022). Our findings contribute to this literature as they demonstrate that with time, the direction of the relationship can flip even within the same population.

Component 3 was the most dramatic in terms of functional shifts, which were observed in the study and repeatedly alternated between strong protection and a strong risk throughout all the 5 waves. In the initial phase of the pandemic in 2020, reliance on quality information and emotional processing was the strongest negative factor (*r* = −0.225). This short-term psychological resource decreased uncertainty and strengthened cognitive control, which allowed individuals to cope with early chaos (Grupe and Nitschke, 2013; Chao et al., 2020).

Interestingly, only a year later, in 2021, this factor flipped to the most significant threat (*r* = 0.238), representing a significant informational burden. Constant monitoring of crisis-related news became cognitively taxing under the condition of unresolved uncertainty, which is consistent with headline stress disorder and the evidence demonstrating that excessive media consumption of COVID-19-related news leads to increased anxiety and depression (Dong and Zheng, 2020; Bendau et al., 2021; Hoyt et al., 2022). In 2022, the effect neutralized (*r* = −0.007, ns), which probably resulted in habituation to the effect or strategic disengagement with media, as it had been observed in long-term lockdown studies where initial effects attenuated over time (Grygarová et al., 2022).

In 2023 (*r* = −0.271), trust re-emerged as a powerful protector, potentially due to the renewed value people have placed on authoritative sources amid the growing war-related disinformation campaigns. Finally, persistent vigilance (regardless of the quality of the source) returned to risk (*r* = 0.150) by 2025 (Epel et al., 2018; Lee-Won et al., 2025). This oscillatory movement is consistent with the perseverative cognition hypothesis (Ottaviani, 2018) where recurrent rumination on uncontrollable threats maintains physiological arousal without a resolution (Nolen-Hoeksema, 1991; Hernández-Posadas et al., 2023). With time, the cumulative stress-response system of wear and tear diminished the adaptability of the stress-response system, turning an initial asset into an illness liability.

Component 2 exhibited the largest significant functional change. It had a small yet statistically significant negative effect in 2020 (*r* = −0.126) that may be likened to an initial phase of the pandemic, where shared digital experiences created a sense of belonging and alleviated loneliness during the first days of the pandemic, when it was an acute phenomenon. This finding aligns with the literature that online communication may enhance wellbeing through social connectivity, especially in situations where physical interactions are restricted (Best et al., 2014; Berry et al., 2017). Although the effect became neutral in 2021 (*r* = −0.030, ns), in 2022 Component 2 acquired a powerful buffering effect (*r* = −0.182), as an active social shield, reducing depressive symptoms (Brown and Kuss, 2020), which confirms that social media can be used as support in times of collective crisis. This correlates with the Russian invasion of Ukraine (February 2022), which suggests that an acute geopolitical crisis may have been followed by the reactivation of the adaptive role of digital communities, an act of providing real-time information sharing, solidarity, and collective comprehension (Brown and Kuss, 2020; Gottschick et al., 2023; Mottola et al., 2023).

By 2023, however, there was a reversal in the directionality of this association, such that it became a statistically significant risk factor (*r* = 0.059). This polarity shift indicates a moment of affective saturation and systemic social exhaustion for the observed population. Researchers have found that social media use is associated with depression (Ahmed et al., 2022; Coyne et al., 2019), and sustained online engagement has been shown to foster social comparisons, surveillance, and FOMO (Elhai et al., 2021; Faelens et al., 2021). Moreover, by 2025, Component 2 had re-emerged as the strongest negative factor in the entire study (*r* = −0.276). Despite prolonged social-interactive exposure becoming a stressor, populations may eventually learn how to leverage digital communities as a source of long-term resilience, as the U-shaped trajectory indicates.

The alteration of the negative to positive, and the reverse, could be attributed to the modification of the rumination process of social media content. Rumination, repetitive, passive thinking about one's distress and its causes (Nolen-Hoeksema, 1991), is more important than other variables in explaining the association between social media use and depression (Ma and Liu, 2026). Early in the crisis, social media use may have involved active, problem-focused engagement that reduced rumination. Passive scrolling and upward social comparison promoted ruminative thinking as the situation worsened, turning originally adaptive participation into a maladaptive loop. The HPA axis and autonomic nervous system are two stress-response systems that are kept chronically active by this ruminative persistence, which is significant with wear and tear and the cumulative allostatic load that shows up as depressive symptoms (Epel et al., 2018; Ottaviani, 2018; Lee-Won et al., 2025). By the time the final wave was conducted, users were using mental strategies to notice and stop repetitive patterns of negative thinking and were thereby able to use social media for interactions that were based on real shared experiences. In this state, the body does not experience the physical wear that results from chronic stress. This explanation aligns with results showing that repetitive negative thinking is the link between the state of media-induced uncertainty and depression (Kesner et al., 2025; Hernández-Posadas et al., 2023).

Component 1 showed no significance during the years 2020 (*r* = −0.032, ns), 2021 (*r* = −0.002, ns) & 2023 (*r* = −0.033, ns), remaining psychologically passive throughout the study. This relative neutrality aligns with meta-analytic results that Economic Reporting and Serious News consumption, in the absence of crisis framing, have weak or inconsistent relationships with mental health outcomes (De Hoog and Verboon, 2020; Boukes and Vliegenthart, 2017). However, in 2022, it emerged as a mild risk factor (*r* = 0.055), indicating that general economic and routine news began to take an emotional toll during the prolonged crisis, a pattern observed following the Russian invasion of Ukraine, where media exposure was associated with distress, anxiety, and vicarious traumatization (Gottschick et al., 2023; Malecki et al., 2023; Kurapov et al., 2023).

By 2025, this factor had become negatively significant (*r* = −0.104). We understand this late-stage protection as a grounding mechanism that may function by stopping rumination and preventing further wear and tear. In contrast to the highly emotional domains, Interactive Content and Readers' Comments (Component 2) and Emotional Impact and Media Stress (Component 3), regular intake of non-crisis, general news may act as a cognitive anchor after years of constant exposure to crises.

Routine news may offer a sense of normality and correlation that enables the brain to break free from ruminative cycles, in contrast to Component 3 (Emotional Impact and Media Stress), which creates uncertainty and encourages perseverative cognition (Kesner et al., 2025). Because it keeps the stress-response system in a low-activation, recovery-permitting condition, low-arousal, predictable material may be the only type of media interaction that is not significant with additional allostatic load from a wear-and-tear standpoint. According to studies on media-induced trauma, not all media exposure is created equal. Routine, low-arousal content may offer a sense of predictability and normalcy, protecting against the cumulative stress of high-arousal crisis monitoring (Trnka and Lorencova, 2020; Pfefferbaum et al., 2014).

The most notable macro-level result is that by 2025, each of the three factors showed a statistically significant association with depression (*p* < 0.001). In previous years, one or two components remained neutral, but the final wave indicates no psychologically inert media behavior. This is a condition of complete integration in which mental health outcomes are constantly negotiated by every aspect of the media environment.

Our hypothesis posits that rumination mediates the impact of media exposure, translating it into physiological and psychological wear and tear across all three trajectories. Rumination is found as the cognitive driver that explains media exposure into the wear and tear (allostatic stress) across all three components. Interactive content and readers' comments are stress activation processes (Component 2) where rumination transforms protective social connections into harmful social comparison and FOMO (Elhai et al., 2021; Ma and Liu, 2026). Rumination supports the perseverative reasoning that turns adaptive surveillance into the perpetual allostatic load associated with emotional effects and media strain (Component 3; Ottaviani, 2018; Hernández-Posadas et al., 2023). This absence of rumination-inducing content with economic reporting and serious news (Component 1) can contribute to explaining why this area eventually offered engagement without long-term stimuli that could be recognized in social or crisis information, leading to comparatively less wear.

A dynamic tension in the psychological base of the population is now established between the grounding of routine (Component 1 as Mild), the relief of sharing (Component 2 as Moderate), and the stress of watching (Component 3 as potential risk). The population no longer draws a line between media and environment, and each act of media has an apparent psychological and physiological meaning. It is precisely this framework that one would expect to be present during chronic, cumulative allostatic load (Epel et al., 2018; Lee-Won et al., 2025). By 2025, the population had not returned to pre-crisis levels. Rather, they had established a higher ground of activation of stress systems, or a new allostatic set point, which is now the new normal.

All three factors are highly correlated with depression in the final wave since in this condition, previously suppressed media habits now bear some allostatic cost. The safeguarding influences that we still experience (Component 2, *r* = −0.276) reflect interaction patterns that reduce new strain and provide compensatory benefits (strong association, social support), instead of having wear and tear.

This result aligns with a growing body of literature demonstrating the relationship between rumination and the association between media use and depression (Hernández-Posadas et al., 2023; Kesner et al., 2025; Ma and Liu, 2026). The change in the interactive content and comments by readers (Component 2) that shifted from negative to at-risk to negative again might be due to the change in the way people view the material in the media over time. Passive, repetitive processing might have been a consequence of long-term exposure; active and meaning-oriented early engagement might have been followed by eventual adaptation through the acquisition of cognitive skills to recognize and disrupt ruminative cycles before they can accrue an increasing burden of allostatic load.

## Conclusion

When it comes to the associations between digital media consumption and depressive symptoms, descriptively, there were significant non-linear shifts across the five consecutive crises. Component 2, which was found to be related to depressive symptoms in a neutral and positive manner in previous years, ultimately was the strongest negative correlate in 2025 (*r* = −0.276, *p* < 0.001), indicating that it may be a possible defense mechanism in the population. The association for component 3 was negative at the beginning, positive during the crisis and later descriptive toward a long-term risk factor. Until it imploded as a possible grounding buffer in the final wave, Component 1 was neutral. The results were not a simple dichotomy of “media is good” vs. “media is bad,” but instead supported a wear-and-tear perspective, which proposes that rumination serves as the cognitive mechanism linking media exposure and allostatic load. The same behavior can be seen as adaptive or maladaptive depending on the crisis stage. In 2025, all three components were found to be significantly correlated, highlighting strong associations between media use and the underlying context of mental health. In this model, certain types of engagement with the media may foster rumination processes linked to depressive symptoms, whereas other types may suppress the iterative effects of chronic exposure to crisis.

## Recommendations

Future research must examine whether similar component patterns can be observed in other national settings and whether particular interventions, namely those that reduce allostatic load (e.g., structured media breaks, digital detoxes, periods of rest), and rumination (e.g., cognitive-behavioral therapy, mindfulness training, media literacy programs that train ruminative trigger recognition) can change such patterns. Studies ought to differentiate passive consumption (doomscrolling, thoughtfully selected profile reading, and more) and active use (communicating with close friends, creating content, and engaging in supportive networks) and examine their different relationships with depressive symptoms. Research should look at the effects on mood, self-perception, and worldview of algorithms that support engagement-driven content (such as conflict, extreme fitness, and wealth displays) and how they could be optimized for user wellbeing.

Also, more controlled experimental designs are needed to identify the specific media elements (such as content type, algorithmic curation, and social comparison characteristics) that contribute to negative outcomes and to show causal pathways toward psychological distress.

## Limitation

There are a few limitations that should be noted. First, research has shown that people with depression may use digital media differently. Therefore, reverse causality, depressed people seeking out media or ruminating more on media could be conceivable. Correlations do not imply causation. Additionally, the collection of data was not even across the years. The components of media consumption were not administered in 2024, creating a gap in the longitudinal timeline of 1 year between 2023 and 2025. This omitted wave means that we will not be able to determine whether the changes that we will see in 2025 were gradual or sudden. Sample sizes declined across waves-−2020 (*N* = 2,192), 2021 (*N* = 2,061), 2022 (*N* = 1,707), 2023 (*N* = 1,707)—reflecting a ~22% dropout likely due to pandemic disruptions, geopolitical turmoil, and researcher burnout, before rebounding in 2025 (*N* = 2,072) via renewed recruitment. This loss can contribute to selection bias, as retainers might systematically differ in resilience, level of depression, or media habits from dropouts, whereas re-enrolment leads to cohort effects. Additionally, the study did not account for content-specific exposure to understand patterns, which could have a different effect on psychological outcomes based on narrative consumed by the participant (e.g., misinformation, health-related news, or supportive material).

## Data Availability

The raw data supporting the conclusions of this article will be made available by the authors, without undue reservation.
